# Stigmasterol Alleviates Cutaneous Allergic Responses in Rodents

**DOI:** 10.1155/2018/3984068

**Published:** 2018-07-24

**Authors:** Aaron O. Antwi, David D. Obiri, Newman Osafo, Leslie B. Essel, Arnold D. Forkuo, Clement Atobiga

**Affiliations:** ^1^Department of Pharmacology, Faculty of Pharmacy and Pharmaceutical Sciences, College of Health Sciences, Kwame Nkrumah University of Science & Technology (KNUST), Kumasi, Ghana; ^2^Department of Pathology, Komfo Anokye Teaching Hospital (KATH), Kumasi, Ghana

## Abstract

The therapeutic potential of stigmasterol, a natural steroid alcohol with established immune-modulatory properties, was assessed on allergic cutaneous responses. We examined its suppressive effect on immunoglobulin E (IgE)-mediated active cutaneous anaphylaxis (ACA), compound 48/80 (C48/80)-induced pruritus, and irritant dermatitis induced by 12-O-tetradecanoylphorbol-13-acetate (TPA). Stigmasterol at 10–100 mg/kg significantly inhibited ACA with reduction in reaction area and concentration of the extravasated Evans blue dye. Given at 50 and 100 mg/kg, stigmasterol significantly inhibited C48/80-induced scratching behaviour when compared to saline-treated C48/80-injected control. Skin histopathology of injected sites confirmed that stigmasterol reduced mast cell trafficking and degranulation associated with C48/80-induced pruritus. Stigmasterol controlled inflammatory features such as ear skin oedema and neutrophilia and also reduced serum levels of TNF*α* induced by topical application of TPA. Epidermal layer thickening and inflammatory cell infiltration of ear skin tissue were significantly reduced by stigmasterol. Taken together, stigmasterol demonstrates significant potential as a molecule of interest in allergic skin disease therapy.

## 1. Introduction

The skin is a large complex immunological organ that plays various roles in sensory functions, temperature control, and host protection. Thus, it is critical in the defense against pathogens and allergic responses [1]. A complex interplay of barrier cells, resident and migratory immune cells, and an array of inflammatory mediators makes the skin highly effective in its functions [2, 3]. The large surface area and exposure to the environment however make the skin particularly vulnerable to environmental allergens, chemicals, and pollutants [4]. It is also one of the most affected organs in adverse drug reactions [5].

Overelaborate immune responses to these agents as well as ‘unchecked' internal mechanisms account for a range of skin disorders including atopic dermatitis, contact dermatitis, urticaria, angioedema, and psoriasis [6, 7]. These disorders are collectively known as allergic skin diseases (ASD). Central to the pathogenesis of these allergic skin diseases are resident structural cells such as keratinocytes, skin mast cells, and antigen presenting cells such as Langerhans cells and dendritic cells [8, 9]. After disruption of the epidermal layer and subsequent entry of allergens or irritants, these innate cells initiate a variety of early immune responses [10, 11]. Consequently, activities of proinflammatory cytokines such as tumor necrosis factor alpha (TNF*α*) and interleukin-1 (IL-1), chemoattractants like C-X-C motif chemokine ligand 8 (CXCL8, IL-8), and the migration of inflammatory cells into skin tissue promote a hyperinflammatory state characterized by urticaria, eczema, pruritus, and other dermatological changes [12, 13]. Skin mast cells in particular contribute to allergic skin diseases* via* both immunoglobulin E (IgE)-dependent and non-IgE-dependent mechanisms. The degranulation of the mast cell with the subsequent release of stored and* de novo* synthesized vasoactive amines, proinflammatory cytokines, and lipid mediators contribute to the underlying inflammation and clinical manifestations of most allergic skin diseases [14].

Pruritus is a common symptom in allergic skin disorders. Controlling scratching behaviour promotes faster healing of the epithelial barrier, enhances patient comfort, and prevents secondary infections [1, 15]. While the release of pruritogens such as histamine and serotonin from mast cells is implicated in itch responses in skin diseases, reports on the effectiveness of antihistamines are conflicting and largely inconclusive [16, 17]. Several research findings have also demonstrated the ineffectiveness of mast cell stabilizers in controlling pruritus [18–20], thus stressing the involvement of other pathways. Alternatives to the current treatment of allergic skin diseases have become the subject of recent studies [21, 22]. A number of studies have reported effectiveness for both topically applied and systemically administered plant-derived and other naturally occurring compounds [23–25]. Phytosterols in general have been shown to have significant modulatory effect on inflammatory gene transcription [26], proinflammatory mediator release [27], and generation of reactive oxygen species [28] and are therefore potential candidates for allergic skin disease therapy.

Stigmasterol, a steroid alcohol, one of such phytosterols that is found in a number of medicinal plants, vegetables, and nuts, has acclaimed and proven immune-modulatory properties either alone or as a component of phytosterol mixtures [29–31]. We have previously shown that stigmasterol attenuates both innate and adaptive immune responses. We demonstrated its inhibitory effects on inflammatory cell recruitment, allergen and non allergen-induced oxidative stress, and expressions of IgE and vascular cell adhesion molecule-1 (VCAM-1), respectively [32, 33].

The focus of this present study is to assess the suppressive effect of stigmasterol on cutaneous anaphylaxis; compound 48/80-induced pruritic responses and TPA-induced skin inflammation; models that reflect IgE and non-IgE-dependent mechanisms.

## 2. Materials and Methods

### 2.1. Materials

#### 2.1.1. Chemicals and Reagents

Stigmasterol (95 %), dexamethasone, 12-O-tetradecanoylphorbol-13-acetate (TPA), compound 48/80 (C2313), polyethylene glycol (PEG), Evans Blue dye, and ketotifen fumarate were obtained from Sigma Aldrich (St. Louis, USA). Bovine Serum Albumin (BSA) was purchased from PAA Laboratories (Marburg, Germany). Phosphate Buffered Saline (PBS) was procured from Gibco (Karlsruhe, Germany). Rat TNF*α* ELISA quantification kit was obtained from MLBio Biotechnology Company Limited (Shanghai, China).

#### 2.1.2. Animals

ICR mice (25-30 g) and Wistar rats (100-120 g) of either sex were purchased from Noguchi Memorial Institute for Medical Research (NMIMR), Legon, Ghana, and kept under standard temperature and humidity conditions (temperature 23 ± 2°C with a 12 h light-dark cycle) at the animal house facility of the Department of Pharmacology, Faculty of Pharmacy and Pharmaceutical Sciences, KNUST. Experimental animals were allowed access to commercial chow and distilled water* ad libitum*. All protocols used in this study were approved by the Faculty of Pharmacy and Pharmaceutical Sciences Ethics Committee and animal handling was done in compliance with Animal Welfare Regulations (USDA 1985; US Code, 42 USC § 289d) and the Public Health Service Policy on Humane Care and Use of Laboratory Animals (PHS 2002).

### 2.2. Methods

#### 2.2.1. Active Cutaneous Anaphylaxis

ICR mice (25-30 g, n = 5) were immunized with 100 *μ*l BSA (0.05 mg/ml,* s.c.*) on the start day of the experiment. Immunization was repeated with a booster dose of 100 *μ*l BSA (0.02 mg/ml* s.c.*) on day 14. Mice received an injection of 200 *μ*l Evans blue dye (1 %  w/v)* via* the tail vein on day 21 and were randomized into 6 groups for the following treatments intraperitoneally:

Group I: naïve control-normal saline (0.9 %  w/v) (5 ml/kg).

Group II: vehicle control-polyethylene glycol, PEG (50 %  w/v) (5 ml/kg).

Group III: sodium cromoglycate (10 mg/kg).

Group IV – VI: stigmasterol (10, 50, and 100 mg/kg), respectively.

Under mild anaesthesia sensitized mice were challenged by inoculation with 100 *μ*l BSA (0.1 mg/ml) on both pinnae using a 21-gauge hypodermic needle 1 h after drug treatment. Naïve animals (n = 5) were sham-sensitized with 100 *μ*l normal saline and challenged with 100 *μ*l PBS only. All mice were euthanized 30 min after the antigen challenge. Evans blue dye was extracted from skin tissue by a method described by Je et al. [34]. Each pinna was placed in a stoppered glass tube containing 1 ml KOH (1M) and kept overnight at 37°C. A 4 ml mixture of phosphoric acid and acetone (5:13) was added to each tube and shaken vigorously. The resulting mixture was centrifuged (C 257-120, Wagtech International, UK) at 1000 rpm for 15 min at 25°C. Absorbance of extracted dye from each pinna was measured at 620 nm with a microplate reader (Synergy HI Multi-Mode, BioTek Technologies, Winooski, USA). Respective concentrations were interpolated from the standard calibration curve developed from serial dilutions of pure Evans blue dye.

#### 2.2.2. Compound 48/80-Induced Pruritus

ICR mice (20-30 g, n = 5) were placed in Perspex observation chambers for 1 h to acclimatize before the experiment and randomized into 6 treatment groups as follows:

Group I: naïve control- normal saline (5 ml/kg,* i.p*.).

Group II: vehicle control-polyethylene glycol, PEG (50 %  w/v) (5 ml/kg,* i.p*.).

Group III: ketotifen fumarate (10 mg/kg,* p.o*.).

Groups IV–VI: stigmasterol (10, 50, and 100 mg/kg,* i.p*.) respectively.

Test mice received subcutaneous injections of 100 *μ*g compound 48/80 (dissolved in 100 *μ*l PBS) at the rostral part of the back 30 min after respective treatments, while naïve mice were given 100 *μ*l PBS only. Immediately after injections, the mice were placed in the observation chambers and itch responses were recorded for 1 h. Scratching behaviour was observed and scored in accordance with the method described by Shafizadeh et al. [35]. Scratching was defined as a motor behaviour in which hind paws were applied repeatedly to the point of injection. A scratching series followed by a minimum of 1 s pause was considered as one scratching bout. Limb movements towards other parts of the body were considered as grooming and were excluded from the score.

Skin sections (2 mm^2^) from the site of injection were subsequently excised, stored in 10 %  w/v buffered formalin, and embedded in paraffin. Sections of 3 *μ*m thickness were cut, deparaffinized, dehydrated, and stained with toluidine blue (1 %  w/v, pH 2.5) for assessment of mast cell proliferation (number of mast cells per square millimeter area of skin tissue) and percentage degranulation (number of degranulated mast cells per total cells in square millimeter area). Mast cell counts were performed by an independent observer. Morphometric analysis of skin area was performed with Image J analysis tool (version 1.50i, Maryland, USA).

#### 2.2.3. TPA-Induced Dermatitis

Wistar rats of either sex (100–120 g, n = 5) were randomly placed in six groups and treated* via* the intraperitoneal route as follows:

Group I: naïve control-normal saline (5 ml/kg).

Group II: vehicle control-polyethylene glycol, PEG (50 %  w/v) (5 ml/kg).

Group III: dexamethasone (3 mg/kg).

Groups IV–VI: stigmasterol (10, 50, and 100 mg/kg) respectively.

Test rats were challenged topically with 20 *μ*g TPA (12-O-tetradecanoylphorbol-13-acetate) dissolved in acetone (20 *μ*g/ 20 *μ*l) and applied on both inner and outer surfaces of each ear while naïve control rats received a topical application of acetone only. Drug treatment and subsequent challenge were repeated in 24 h and 48 h for all groups. Rats were sacrificed by cervical dislocation 5 h after the last TPA or acetone challenge. Both ears of each rat were cut off and weighed. Increase in ear weight compared to control was considered as a measure of oedema. The jugular vein was quickly dissected and the following determinations made.

*(1) Neutrophil Count*. Blood was collected into EDTA tubes for neutrophil count with an automated analyzer (Sysmex KX-21N, Sysmex America Inc., Illinois, USA).

*(2) Serum TNFα*. Blood was collected into sterile gel and clot activator tubes. Sera were separated by centrifugation (C 257-120, Wagtech International, UK) at 2000 rpm for 15 min at 20°C. Aliquots of serum were collected into Eppendorf tubes and stored at -70°C until needed. Serum levels of TNF*α* were quantified using enzyme-linked immunosorbent assay (ELISA) kit according to the instructions prescribed by the manufacturer.

*(3) Histopathology*. Excised ear tissues were stored in 10 %  w/v buffered formalin and embedded in paraffin. Sections of 3 *μ*m thickness were cut, deparaffinized, dehydrated, and stained with haematoxylin and eosin (H & E). The tissue sections were observed for inflammatory changes under light microscope, Leica DM2500 M (Leica Microsystems, Wetzlar, Germany). Three random fields for each skin section of randomly selected animals in each group were examined for leukocyte infiltration, skin, and epidermal layer thickness. Morphometric analyses of skin and epidermal layer thickness were performed with Image J analysis tool.

## 3. Statistics

Data is presented as mean ± standard error of mean (SEM). Data analysis was performed using one-way analysis of variance (ANOVA). Multiple comparisons between treatment groups were done using Dunnett's* post hoc* test. GraphPad for Windows version 6 (GraphPad Prism Software, San Diego, USA) was employed for all statistical analyses.

## 4. Results

### 4.1. Effect of Stigmasterol on Active Cutaneous Anaphylaxis

**Figure 1:**
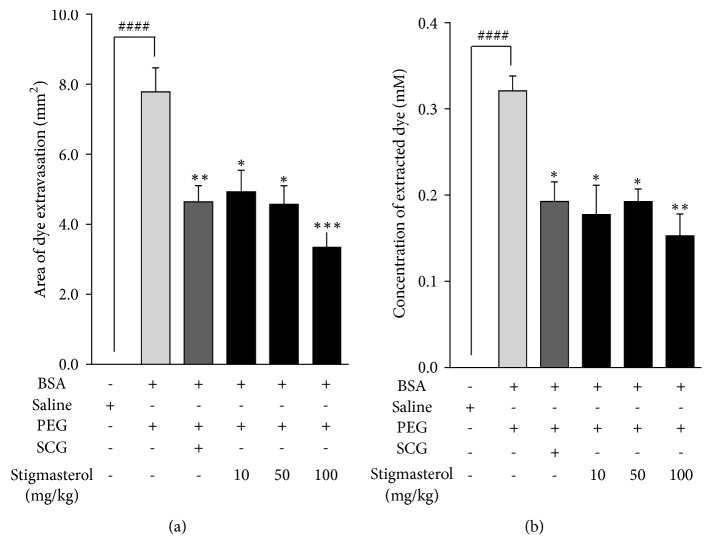
Effect of stigmasterol on active cutaneous anaphylaxis in mice. ICR mice were sensitized with BSA as previously described. Mice were treated with either normal saline (5 ml/kg), polyethylene glycol (5 ml/kg), sodium cromoglycate, SCG (10 mg/kg), or stigmasterol (10, 50, and 100 mg/kg) and challenged for 30 min by inoculation of BSA on each pinna. Pinnae were excised and reaction area was recorded (a). Absorbance of extravasated dye was determined (b). Data is expressed as mean area or mean dye concentration ± SEM (n = 10). ^*∗∗∗*^*P* < 0.001, ^*∗∗*^*P* < 0.01, and ^*∗*^*P* < 0.05 as compared to PEG-treated control. ^###^*P* < 0.001 as compared to saline-treated naïve control using one-way ANOVA followed by Dunnett's* post hoc* test.

Cutaneous challenge with bovine serum albumin induced a local inflammation marked by Evans blue dye extravasation in all the PEG vehicle-treated test mice. A significantly increased mean inflammatory reaction area of 7.79 ± 0.68 mm^2^ was observed in PEG-treated control mice relative to 0.06 ± 0.02 mm^2^ in the non sensitized saline-treated naïve control (Figure 1(a)). Mice pretreated with stigmasterol 30 min before antigen challenge showed markedly reduced active cutaneous anaphylaxis. Mean reaction areas of 4.93 ± 0.62 mm^2^, 4.57 ± 0.53 mm^2^, and 3.36 ± 0.40 mm^2^ were obtained for stigmasterol at 10, 50 and 100 mg/kg, respectively, (Figure 1(a)) representing mean percentage inhibitions of 36.70 ± 7.90 %, 41.29 ± 6.79 %, and 56.88 ± 5.15 %, respectively, of the active cutaneous anaphylaxis. A mean inflammatory reaction area of 4.64 ± 0.46 mm^2^ (Figure 1(a)) was obtained on pretreatment with sodium cromoglycate inhibiting the dye extravasation area by 40.37 ± 5.96 %.

Quantification of the extravasated dye through absorbance measurements presented a similar trend. In the PEG-treated control mice a significant increase in mean concentration of dye to 0.32 ± 0.02 mM was obtained when compared to that for the naïve control of 0.005 ± 0.0008 mM. At the doses of stigmasterol used the concentration of extracted dye were 0.18 ± 0.03 mM, 0.19 ± 0.01 mM and 0.15 ± 0.03 mM, respectively (Figure 1(b)). Thus stigmasterol offered 44.74 ± 10.62 %, 40.08 ± 4.59 %, and 52.38 ± 7.87 % reductions in the concentration of the extravasated dye, respectively, relative to PEG-treated control group at 10-100 mg/kg. Sodium cromoglycate reduced extravasated dye concentration to 0.19 ± 0.02 mM representing a 39.95 ± 7.00 % inhibition compared to the PEG-treated control group (Figure 1(b)).

### 4.2. Compound 48/80-Induced Pruritus

#### 4.2.1. Effect of Stigmasterol on Compound 48/80-Induced Scratching Behaviour

**Figure 2:**
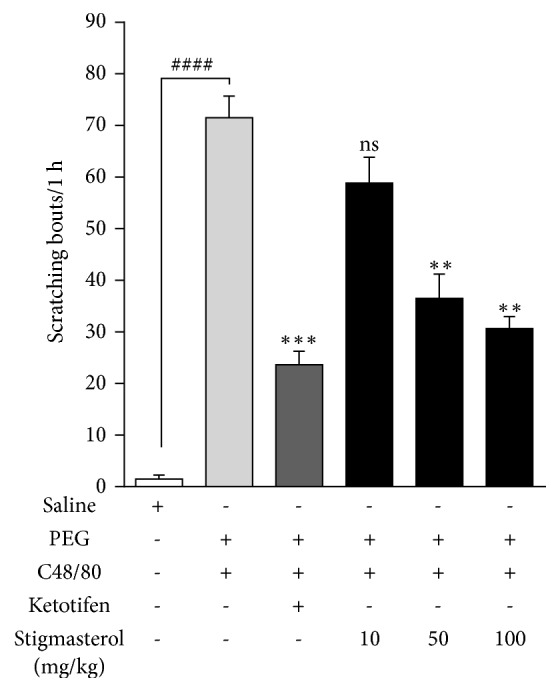
Effect of stigmasterol on C48/80-induced scratching behaviour in mice. Male ICR mice received either normal saline (5 ml/kg), polyethylene glycol PEG (5 ml/kg), ketotifen (10 mg/kg), or stigmasterol (10, 50, and 100 mg/kg). Test mice received C48/80 (100 *μ*g* s.c.*) in the rostral back region and scratching behaviour observed for 1 h as previously described. Data is expressed as mean scratching bout/ 1 h ± SEM (n = 5). ^*∗∗∗*^*P* < 0.001; ^*∗∗*^*P* < 0.01; ns is not significant as compared to PEG-treated control. ^####^*P* < 0.0001 as compared to saline-treated naïve control using one-way ANOVA followed by Dunnett's* post hoc* test.

Intense scratching behaviour was induced by subcutaneous injection of C48/80 in all test mice. Itch responses such as hind limb scratching, biting, and front limb rubbing of mainly the injected site (rostral back) and other parts of the body were observed in injected mice in which only specific hind limb rostral scratching sequences were recorded for consistency and to differentiate from normal grooming. Mean scratching bouts of 71.50 ± 4.22 were recorded in PEG-treated C48/80-injected control mice compared to mean counts in saline-treated naïve mice of 1.50 ± 0.76 (Figure 2). Stigmasterol significantly reduced the mean scratching bouts to 36.50 ± 4.72 and 30.67 ± 2.28, thereby reducing the scratching intensity by 48.95 ± 6.61 % and 57.11 ± 3.18 %, respectively, when administered at 50 and 100 mg/kg in comparison with the C48/80-injected control mice. No significant inhibition was observed at 10 mg/kg with mean scratching bouts of 58.83 ± 5.04 (Figure 2). Ketotifen significantly reduced the mean scratching bouts to 23.67 ± 2.57 (Figure 2) representing 66.90 ± 3.59 % inhibition of scratching intensity.

#### 4.2.2. Effect of Stigmasterol on Skin Mast Cell Proliferation and Degranulation

**Figure 3:**
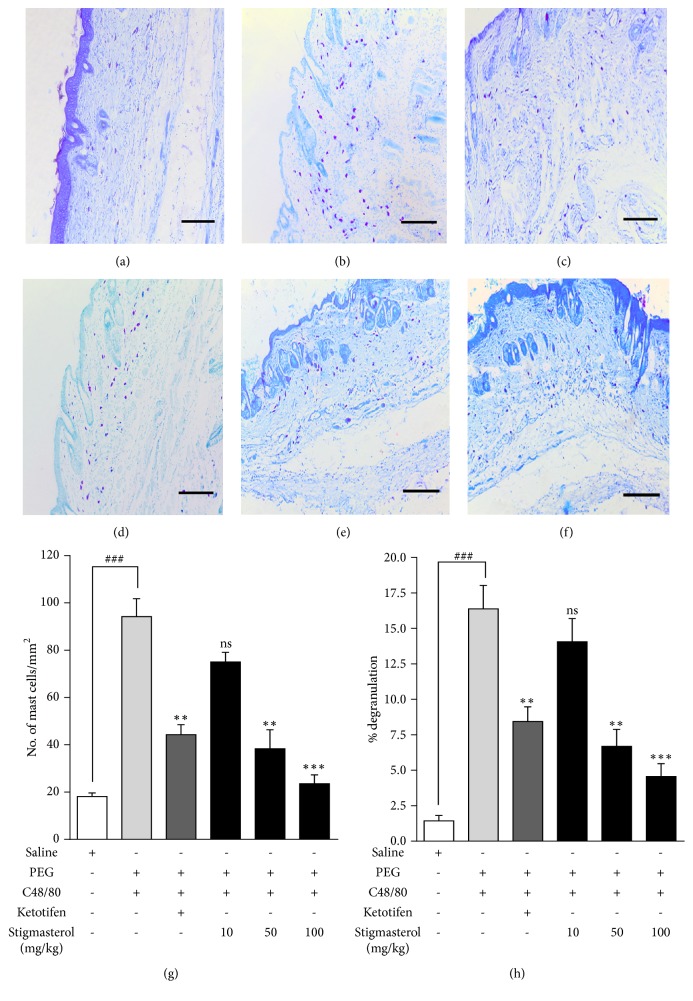
Effect of stigmasterol on C48/80-induced mast cell proliferation and degranulation. Male ICR mice received either normal saline (5 ml/kg), polyethylene glycol, PEG (5 ml/kg), ketotifen (10 mg/kg), or stigmasterol (10, 50, and 100 mg/kg). Test mice received C48/80 (100 *μ*g,* s.c*.). Skin sections from injected sites were excised, fixed, and embedded in paraffin. 3 *μ*m sections were stained with toluidine blue 1 h later to assess mast cell distribution in naïve (a), polyethylene glycol, PEG (b), ketotifen (c), and 10-100 mg/kg stigmasterol-treated groups (d–f). Mast cells were quantified (g and h). Data is expressed as mean mast cell number (cells/mm^2^) ± SEM and mean % degranulation ± SEM. ^*∗∗∗*^*P* < 0.001; ^*∗∗*^*P* < 0.01; ns is not significant as compared to PEG-treated C48/80-injected group. ^###^*P* < 0.001 as compared to saline-treated naïve control group using one-way ANOVA followed by Dunnett's* post hoc* test. Micron bar represents 300 *μ*m.

Skin sections from the rostral back of naïve ICR mice showed normal skin histology. Toluidine blue staining revealed sparse mast cell distribution within the dermal region. No mast cell clusters occurred and only limited mast cell degranulation was observed (Figure 3(a)). Skin sections from PEG-treated control mice were however characterized by profound proliferation of mast cells within the dermal region with several mast cell clusters and incidents of degranulation (Figure 3(b)). These features were unchanged when mice were treated with 10 mg/kg stigmasterol (Figure 3(d)). Incidents of mast cell accumulation and degranulation were observed to be reduced in stigmasterol 50 and 100 mg/kg treated mice (Figures 3(e) and 3(f)) as well as in ketotifen treated mice (Figure 3(c)). The number of mast cells per millimeter square area of skin tissue, a measure of mast cell distribution, showed stigmasterol significantly reduced mast cell proliferation at the injected site. In the naïve mice, a mean mast cell number per millimeter square area of skin of 18.13 ± 1.48 cells/mm^2^ was significantly increased fivefold to 94.24 ± 7.53 cells/mm^2^ when PEG-treated control mice were injected with C48/80 (Figure 3(g)). This mean mast cell number was significantly reduced to 38.31 ± 8.07 cells/mm^2^ and 23.56 ± 3.72 cells/mm^2^ representing 59.35 ± 8.56 % and 75.00 ± 3.95 % inhibition of mast cell proliferation by treatment with 50 and 100 mg/kg stigmasterol, respectively. Given at 10 mg/kg, stigmasterol reduced the mean mast cell number per millimeter square area of skin to 75.06 ± 4.08 causing an inhibition of 24.94 ± 5.02 % in mast cell proliferation albeit insignificant when compared to the PEG-treated C48/80-injected control. On the other hand, ketotifen treatment recorded a mean of 44.31 ± 4.21 mast cells/mm^2^ (Figure 3(g)) which represented a 52.98 ± 4.47 % significant reduction compared to PEG-treated C48/80-injected control. The percentage degranulation in the saline-treated naïve control mice was calculated to be 1.44 ± 0.38 % which was significantly increased to 16.38 ± 1.66 % in the PEG-treated C48/80-injected control mice (Figure 3(h)). Treatment with 50 and 100 mg/kg stigmasterol significantly reduced the percentage degranulation, respectively, to 6.69 ± 1.19 % and 4.56 ± 0.90 % when compared to the PEG-treated C48/80-injected control. Thus, respectively, 59.17 ± 7.26 % and 72.15 ± 5.49 % inhibitions in mast cell degranulation were obtained by treatment with 50 and 100 mg/kg of stigmasterol. However, given at 10 mg/kg stigmasterol reduced the mean percentage degranulation to 14.06 ± 1.63 (Figure 3(h)) causing a suppression of 24.94 ± 5.02 % in mast degranulation albeit insignificant when compared to the PEG-treated C48/80-injected control. In the case of ketotifen, the mast cell percentage degranulation was reduced to 8.44 ± 1.04 % compared to the test control, thereby stabilizing the mast cell significantly by 48.49 ± 6.33 %.

### 4.3. TPA-Induced Dermatitis

#### 4.3.1. Effect of Stigmasterol on Ear Oedema

**Figure 4:**
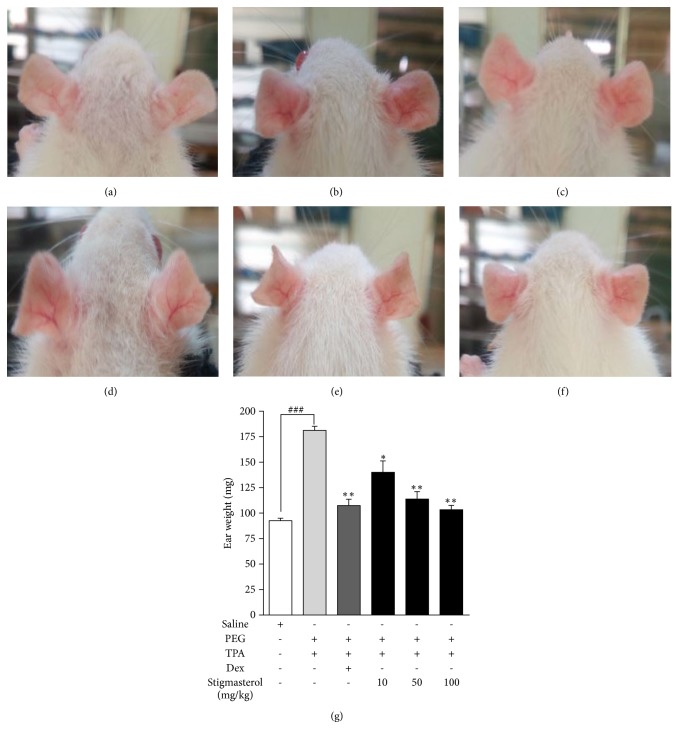
Effect of stigmasterol on TPA-induced ear oedema. Rats received either normal saline (5 ml/kg), polyethylene glycol, PEG (5 ml/kg), dexamethasone, Dex (3 mg/kg), or stigmasterol (10, 50 and 100 mg/kg). Test rats received a topical application of 20 *μ*g TPA dissolved in acetone on each ear daily for 3 days while naïve rats were challenged with acetone only. 5 h after the last TPA or acetone challenge rats were sacrificed and both ears excised to assess oedema in naïve (a), polyethylene glycol, PEG (b), dexamethasone (c), and 10-100 mg/kg stigmasterol-treated groups (d–f). The ear weights were indexed a measure of TPA-induced oedema (g). Data is expressed as mean ear weight (n = 10) ± SEM. ^*∗∗*^*P* < 0.01 and ^*∗*^*P* < 0.05 as compared to PEG-treated TPA-challenged control. ^###^*P* < 0.0001 as compared to saline-treated naïve control using one-way ANOVA followed by Dunnett's* post hoc* test.

Topical application of TPA for 3 days induced significant inflammation in rat ear skin observed as significant increase in ear thickness, dilation of auricular blood vessels, and erythema in PEG-treated TPA-challenged rats (Figure 4(b)) when compared to the naïve acetone-challenged rats (Figure 4(a)). Stigmasterol at 10, 50, and 100 mg/kg caused a significant decrease in these inflammatory features (Figures 4(d)–4(f)). Similarly, inflammation was also significantly suppressed by dexamethasone (Figure 4(c)). Ear weights were indexed as a measure of TPA-induced oedema in ear skin. Mean ear weight of 181.3 ± 3.98 mg recorded for the PEG-treated TPA-challenged rats measured a 1.96-fold increase of 92.50 ± 2.50 mg for the naïve control (Figure 4(g)). TPA-induced increase in ear weight was significantly reduced by both stigmasterol and dexamethasone. Mean ear weights of 140.71 ± 8.52 mg, 113.80 ± 7.34 mg, and 103.34 ± 4.22 mg (Figure 4(g)) representing percentage inhibitions in ear oedema of 21.86 ± 7.46 %, 37.26 ± 4.03 %, 43.00 ± 2.32 %, respectively, compared to PEG-treated TPA-challenged control were obtained in the stigmasterol 10, 50, and 100 mg/kg treated rats. In the group that received dexamethasone a mean ear weight of 107.51 ± 6.23 mg (Figure 4(g)) causing a 40.71 ± 3.42 % inhibition of ear oedema was obtained.

#### 4.3.2. Effect of Stigmasterol on Neutrophil Count

**Figure 5:**
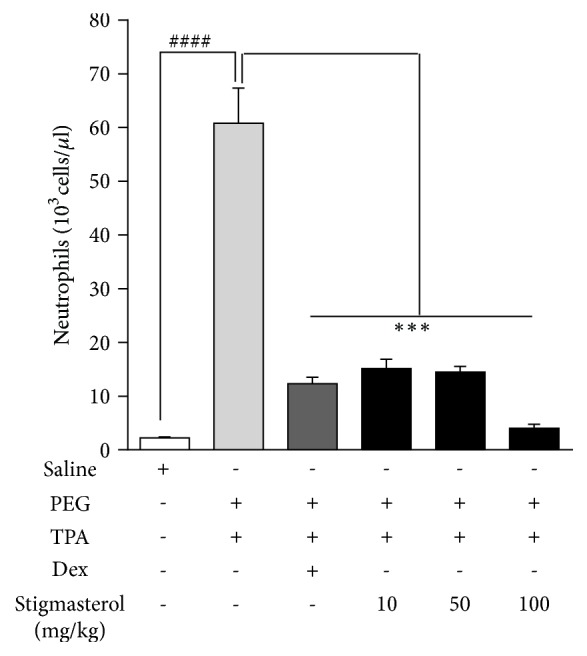
Effect of stigmasterol on TPA-induced neutrophilia. Rats received either normal saline (5 ml/kg), polyethylene glycol, PEG (5 ml/kg), dexamethasone, Dex (3 mg/kg), or stigmasterol (10, 50, and 100 mg/kg). Test rats received a topical application of 20 *μ*g TPA dissolved in acetone on each ear daily for 3 days while naïve rats were challenged with acetone only. 5 h after the last TPA or acetone challenge rats were sacrificed and blood collected into EDTA tubes for neutrophil count. Data is expressed as mean neutrophil count (10^3^ cells/*μ*l) ± SEM. ^*∗∗∗*^*P* < 0.001 as compared to PEG-treated TPA-challenged control. ^####^*P* < 0.0001 as compared to saline-treated naïve control using one-way ANOVA followed by Dunnett's* post hoc* test.

Blood neutrophil count was determined to be 2.25 ± 0.17 x 10^3^ cells/*μ*l in saline-treated naïve control rats (Figure 5). TPA challenge induced severe neutrophilia in the PEG-treated TPA-challenged control with neutrophil cell count of 60.80 ± 6.54 x 10^3^ cells/*μ*l (Figure 5). This represented a 60-fold increase in the count obtained in naïve rats. Stigmasterol at all doses used inhibited this TPA-induced neutrophilia. Treatment with stigmasterol at 10, 50, and 100 mg/kg recorded 15.12 ± 1.77 x 10^3^ cells/*μ*l, 14.47 ± 1.07 x 10^3^ cells/*μ*l, and 4.00 ± 0.77 x 10^3^ cells/*μ*l (Figure 5) representing 75.13± 2.90 %, 76.20 ± 1.75 %, and 93.41 ± 1.27 % inhibition of neutrophil count relative to PEG-treated TPA-challenged control. Similarly, dexamethasone exhibited significant inhibition of neutrophilia. Rats treated with dexamethasone had a mean neutrophil count of 12.33 ± 1.21 x 10^3^ cells/*μ*l (Figure 5) representing 79.73 ± 1.99 % inhibition of TPA-induced neutrophilia.

#### 4.3.3. Effect of Stigmasterol on Serum Levels of TNF*α*

**Figure 6:**
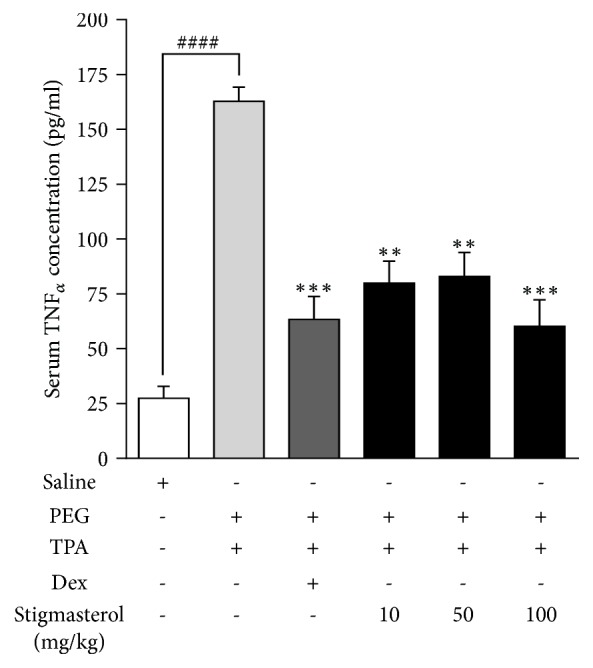
Effect of stigmasterol on TPA-induced increase in serum concentration of TNF*α*. Rats received either normal saline (5 ml/kg), polyethylene glycol, PEG (5 ml/kg), dexamethasone, Dex (3 mg/kg), or stigmasterol (10, 50, and 100 mg/kg). Test rats received a topical application of 20 *μ*g TPA dissolved in acetone on each ear daily for 3 days while naïve rats were challenged with acetone only. 5 h after the last TPA or acetone challenge rats were sacrificed and blood collected. Serum concentration of TNF*α* was quantified with sandwich ELISA. Data is expressed as TNF*α* concentration (pg/ml) ± SEM (n = 5). ^*∗∗∗*^*P* < 0.001; ^*∗∗*^*P* < 0.01 as compared PEG-treated TPA-challenged control. ^####^*P* < 0.001 as compared to saline-treated naïve control using one-way ANOVA followed by Dunnett's* post hoc* test.

TPA challenge caused significant increase in serum level of TNF*α* in the PEG-treated control. Mean TNF*α* concentration in serum collected 5 h after the last TPA challenge was 162.90 ± 6.33 pg/ml, a 6-fold increase above naïve mean levels of 27.40 ± 5.49 pg/ml (Figure 6). Both stigmasterol and dexamethasone treatments suppressed TPA-induced increase in serum TNF*α*. Treatment with 10, 50, and 100 mg/kg of stigmasterol reduced mean serum TNF*α* levels to 79.95 ± 10.00 pg/ml, 82.92 ± 10.98 pg/ml, and 60.21 ± 12.02 pg/ml (Figure 6) indicating 50.92 ± 6.14 %, 49.10 ± 6.74 %, and 63.04 ± 7.38 % inhibitions, respectively, compared to PEG-treated TPA-challenged control. Serum TNF*α* concentration of 63.00 ± 10.53 pg/ml was obtained in the dexamethasone-treated mice (Figure 6) indicating a 61.14 ± 6.47 % inhibition compared to PEG-treated control group.

#### 4.3.4. Histopathology

**Figure 7:**
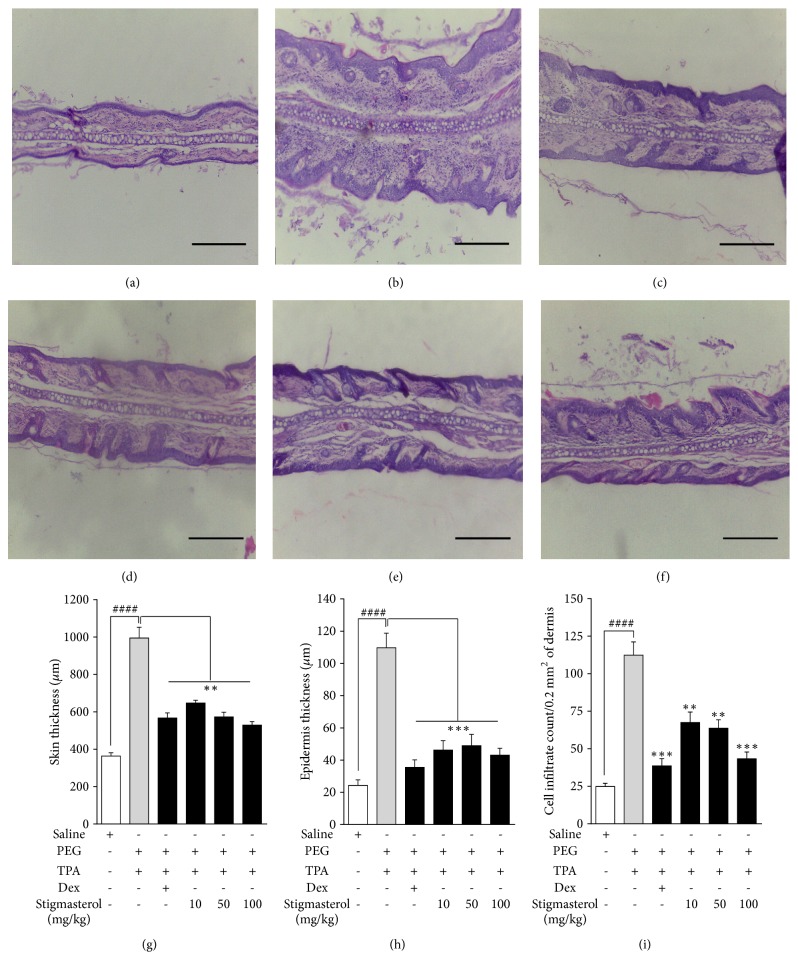
Effect of stigmasterol on TPA-induced dermatitis. Rats received either normal saline (5 ml/kg), polyethylene glycol, PEG (5 ml/kg), dexamethasone, Dex (3 mg/kg), or stigmasterol (10, 50, and 100 mg/kg). Test rats received a topical application of 20 *μ*g TPA dissolved in acetone on each ear daily for 3 days while naïve rats were challenged with acetone only. 5 h after the last TPA or acetone challenge rats were sacrificed and ears excised. 3 *μ*m thickness of skin sections was stained with H & E and observed for histopathological changes in naïve (a), polyethylene glycol, PEG (b), dexamethasone (c), and 10-100 mg/kg stigmasterol-treated animals (d–f) and parameters of skin damage quantified (g–i). Data is expressed as mean skin thickness (*μ*m) (n = 12) ± SEM, mean epidermis thickness (*μ*m) (n = 12) ± SEM, and mean cell infiltrate per field (n = 12) ± SEM. ^*∗∗∗*^*P* < 0.001; ^*∗∗*^*P* < 0.01 as compared to PEG-treated TPA-challenged control. ^###^*P* < 0.0001 as compared to saline-treated naïve control using one-way ANOVA followed by Dunnett's* post hoc* test. Micron bar represents 500 *μ*m.

Skin sections from the saline-treated naïve control animals showed normal skin structure and cell distribution with no signs of hyperplasia of epidermal layer. No sign of oedema was observed in the dermis (Figure 7(a)). TPA induced histological changes consistent with severe inflammation. Skin sections from PEG-treated TPA-challenged rats showed significant distortions in skin architecture. Epidermal hyperplasia with proliferation of epidermal keratinocytes and thickening of dermis contributed to an overall thickened skin section. The dermis was densely populated with clusters of polymorphonuclear leukocytes and lymphocyte infiltrates (Figure 7(b)). Moderate cellular infiltrates and suppressed thickening compared to PEG-treated TPA-challenged rat ear skin sections were observed in stigmasterol 10–100 mg/kg (Figures 7(d)–7(f)) and dexamethasone-treated rats (Figure 7(c)). Indices of inflammatory damage such as skin thickness, epidermal hyperplasia, and inflammatory cell infiltration of dermis were quantified. Skin thickness of PEG-treated TPA-challenged rats measured a mean of 995.00 ± 57.49 *μ*m corresponding to a 173.35 % increase in mean thickness in rat ear skin of the naïve animals which measured 364.00 ± 17.30 *μ*m (Figure 7(g)). Mean thickness in rat ear skin of 647.60 ± 15.04 *μ*m, 573.11 ± 25.29, and 529.40 ± 18.78 *μ*m, respectively, was obtained with stigmasterol 10, 50, and 100 mg/kg, thereby inhibiting the TPA-induced skin thickness by 34.91 ± 1.51 %, 42.41 ± 2.54 %, and 46.80 ± 1.89 %, respectively. Dexamethasone significantly reduced the skin thickness of PEG-treated TPA-challenged rats to 566.70 ± 27.47 *μ*m giving a 43.04 ± 2.76 % inhibition of skin thickness (Figure 7(g)). A similar trend was observed for the epidermal hyperplasia assessment. The average thickness of the epidermis, a measure of epidermal hyperplasia, was found to be 24.28 ± 3.47 *μ*m in naïve rats (Figure 7(h)). Topical TPA challenge induced a 351.81 % increase in thickness of the epidermal layer in the PEG-treated control group with a mean thickness of 109.70 ± 9.1 *μ*m. Stigmasterol at 10, 50, and 100 mg/kg caused significant suppressive effects with average thickness of the epidermis of 46.20 ± 5.85 *μ*m, 48.91 ± 7.02 *μ*m, and 43.04 ± 4.30 *μ*m causing inhibitions of epidermal layer thickening by 57.89 ± 5.34 %, 55.41 ± 6.40 %, and 60.77 ± 3.92 %, respectively. Similarly, in the dexamethasone-treated rats, mean epidermis thickness of 35.47 ± 4.67 *μ*m (Figure 7(h)) representing 67.67 ± 4.26 % inhibition compared to PEG-treated control group was obtained. Quantification of cell distribution in dermal regions of naïve mice recorded a mean cell (polymorphonuclear leukocytes and lymphocytes) count of 24.84 ± 2.19 / 0.2 mm^2^ of dermis (Figure 7(i)). Skin sections of TPA-induced PEG-treated rats had a mean inflammatory cell population of 112.40 ± 8.80 cells/mm^2^ (Figure 7(i)) representing a 352.50 % increase in the count obtained for the naïve control. Stigmasterol 10, 50, and 100 mg/kg gave cell counts of 67.53 ± 6.91 cells/mm^2^, 63.71 ± 5.61 cells/mm^2^, and 43.33 ± 4.52 cells/mm^2^ (Figure 7(i)), thereby reducing TPA-induced cell infiltration by 39.92 ± 6.15 %, 43.32 ± 4.99 %, and 61.45 ± 4.02 %, respectively. In the dexamethasone-treated rats mean cell count of 38.64 ± 4.74 cells/mm^2^ (Figure 7(i)) representing an inhibition of 65.62 ± 4.22 % was obtained.

## 5. Discussion

The effects of stigmasterol on selected allergic cutaneous responses were investigated in this present study. We assessed the therapeutic potential of stigmasterol in antigen-induced cutaneous anaphylaxis, an IgE-dependent mechanism and compound 48/80-induced pruritus as well as TPA-induced irritant dermatitis which are non-IgE-dependent mechanisms.

The cutaneous antigen challenge of previously sensitized mice triggers mast cell activation and degranulation, release of vasoactive mediators, increase in vascular permeability, and dye extravasation. Mast cell stabilizers like sodium cromoglycate, antihistamines (H_1_ receptor blockers), and glucocorticoids given over longer periods have been found to reduce this allergic reaction [36]. In the classic ACA study by Inagaki et al. (1992), mast cell stabilizers and antihistamines were shown to be effective when given in a short period while steroids showed effect when given 8 h prior to allergen challenge. Consistent with this, clobetasol, a glucocorticoid, showed no effect on the wheal and flare response in the first 24 h but did so in a 21-day study period. Thus glucocorticoids are reported to be more effective in the late phase of the allergic response [37–39]. After initial antigen presentation, re-exposure to a cognate allergen leads to cross-linking of immunoglobulin bound to the high affinity Fc*ε*R1 receptors largely expressed on mast cell surfaces and the subsequent activation of the mast cell [40]. The release of preformed mediators, cytokines, lipid mediators, and growth factors subsequent to IgE-mast cell interaction triggers activation of resident innate skin cells, proliferation, and migration of inflammatory cells into skin tissue, pruritus, vascular permeability changes, and oedema formation [41, 42]. Immunoglobulin E (IgE)-mediated mast cell-driven responses are central to allergic skin diseases such as atopic dermatitis. Our findings 30 min after antigen challenge suggest that stigmasterol exerts significant suppressive effect on the early phase of the antigen antibody reaction. We have determined in previous studies the inhibitory effect of stigmasterol on allergen-induced IgE expression [32] and potential mast cell stabilizing effect [unpublished data]. This is consistent with the muted allergic response recorded in this ACA study and earlier reports of antiallergic potential of stigmasterol [43].

We further demonstrated that scratching behaviour induced by compound 48/80 in ICR mice was abated by stigmasterol. Pruritus is a major symptom of dermatological disease which has proven difficult to treat over the years [44]. Incessant scratching further worsens disease prognosis and affects both physical and psychological well-being [45]. Release of histamine and its subsequent stimulation of histaminic-H_1_ receptors is widely reported to be one of the major mechanisms of dermatological itch [46]. Earlier studies by Dunford et al. [20] have also implicated histamine H_2_ and H_4_-receptors in the itch response. Other pruritogens such as serotonin, capsaicin, tryptase, and cannabinoids have all been reported to play important roles* via* a variety of receptors [46]. Perhaps this explains the limited efficacy of antihistamine agents in pruritus. Compound 48/80 is a potent mast cell degranulator widely used in animal models to screen potential antipruritic agents. It acts by directly activating G proteins, stimulating protein tyrosine phosphorylation, and triggering a rise in intracellular calcium [47]. Interestingly, Inagaki et al. [48] demonstrated compound 48/80-induced pruritus in mast cell-deficient mice. The reported ineffectiveness of sodium cromoglycate, a known mast cell stabilizer, in this model and other pruritus studies confirms the possible involvement and importance of other pathways [18, 49]. Consistent with literature however, skin histology of the PEG-treated C48/80-injected control mice showed an increase in mast cell numbers and widespread degranulation at injected site when compared to the saline-treated naïve control. Mast cell proliferation and degranulation have been shown to be critical to the mediation of itch responses in animal studies [19, 50]; both processes we could show were inhibited by stigmasterol.

Topical application of TPA induces skin inflammatory responses consistent with irritant contact dermatitis. In this process, the induction of proinflammatory cytokines and chemoattractants, reactive oxygen species, recruitment of polymorphonuclear leukocytes into skin tissues, and skin histopathology similar to irritant dermatitis is believed to be initiated by activation of protein kinase C, PKC [51]. TPA skin inflammation model is therefore useful in the screening of potential agents in allergic skin diseases [22]. In our study, systemically administered stigmasterol suppressed key local and systemic features of TPA-induced contact dermatitis. Stigmasterol at all doses used reduced ear weights, a measure of oedema, indicating significant inhibition of inflammatory processes. This is consistent with findings in an earlier study by Gomez et al. [52], in which one of several topically applied phytosterol isolates from* Achillea ageratum* that inhibited TPA-induced skin oedema was identified to be stigmasterol. In this paper, while we confirm this action of stigmasterol, we report also that systemic administration of stigmasterol not only reversed oedema but suppressed other key features of the skin inflammatory response. One significant finding was the reduction in serum levels of TNF*α* by stigmasterol. A strong correlation between TPA-induced or clinical contact dermatitis and proinflammatory cytokine activity has been reported widely [53]. TNF*α* which is both an ‘activator' and product of skin cells is crucial in the pathophysiology of allergic skin disease. Its role in activating chemokines such as IL-8 and CCL5 in the early stages of contact dermatitis contributes significantly to infiltration of skin tissue by inflammatory cells. It is therefore not surprising that interventions that control both skin tissue and serum TNF*α* levels have been shown to improve features of TPA-induced dermatitis [54]. Features such as increase in skin thickness, epidermal hyperplasia, and dramatic transmigration of polymorphonuclear leukocytes and lymphocytes which were observed in PEG-treated TPA-challenged control rats were largely suppressed in the stigmasterol-treated rats. Migration of inflammatory cells into skin tissue is a key factor in skin damage in contact dermatitis. Release of chemokines by cells of injured skin encourages the infiltration and trafficking of neutrophils and other inflammatory cells into tissue. Activities of such cells further aggravate the skin injury. Indeed, agents which have shown inhibition of both polymorphonuclear leukocyte activation and chemoattraction into damaged skin tissue have offered significant improvement in allergic skin diseases. Following PKC activation and release of chemokines such as cytokine-induced neutrophil chemoattractant and macrophage inflammatory protein 2, MIP-2 in murine plasma, TPA induces severe systemic neutrophilia and intraepidermal accumulation of neutrophils [55]. Both features of TPA-induced skin inflammation were significantly suppressed on stigmasterol treatment. Perhaps this in addition to reduced serum levels of TNF*α* could account for, albeit partly, the improvement in skin histopathology observed in the stigmasterol-treated rats.

In total, we sought to assess the overall potential of stigmasterol in allergic skin disease (ASD) by studying its effect on aspects of ASD such as antigen antibody reactions, pruritus, and skin inflammation. In each model the choice of reference drug was based on the mechanism of the inducing agent, pharmacology of the control drug, its reported effectiveness in earlier works, and timelines of the study. For example, cromoglycate reduces dye extravasation by preventing allergen-induced mast cell degranulation and subsequent histamine release [36, 56]. Ketotifen controls scratching behaviour due to its mast cell stabilizing and H_1_ antagonist effect [57, 58] while dexamethasone, a glucocorticoid, inhibits expression of proinflammatory cytokines and chemotactic factors induced by TPA application [59].

## 6. Conclusion

Stigmasterol suppressed IgE-mediated vascular permeability changes in allergen-induced cutaneous anaphylaxis. Pretreatment with stigmasterol ameliorated scratching behaviour induced by compound 48/80 possibly due to inhibition of skin mast cell degranulation and proliferation at injected site. Stigmasterol significantly inhibited TPA-induced skin damage. Our data suggests that stigmasterol downregulates cutaneous allergic responses in rodents through suppression of neutrophil accumulation in blood, infiltration of leukocytes into skin tissue, and reduced serum levels of TNF*α*. Stigmasterol therefore holds great potential in allergic skin disease therapy.

## Data Availability

This study forms part of a larger study on stigmasterol leading to the award of a Doctor of Philosophy, Ph.D. Data arising from this particular study are contained within the manuscript. All data shall be deposited with the Research Repository of the Kwame Nkrumah University of Science & Technology, Kumasi, Ghana. Access to these data will be considered by the authors upon request.
